# A Multi Comparison of 8 Different Intraocular Lens Biometry Formulae, Including a Machine Learning Thin Lens Formula (MM) and an Inbuilt Anterior Segment Optical Coherence Tomography Ray Tracing Formula

**DOI:** 10.3390/vision8030049

**Published:** 2024-08-28

**Authors:** Richard N. McNeely, Katherine McGinnity, Stephen Stewart, Emmanuel Eric Pazo, Salissou Moutari, Jonathan E. Moore

**Affiliations:** 1 Cathedral Eye Clinic, Belfast BT1 2LS, UK; 2 Department of Ophthalmology, Belfast Health and Social Care Trust, Belfast BT12 6BA, UK; 3 Tianjin Key Laboratory of Retinal Functions and Diseases, Tianjin Branch of National Clinical Research Center for Ocular Disease, Eye Institute and School of Optometry, Tianjin Medical University Eye Hospital, Tianjin 300384, China; 4 Mathematical Sciences Research Centre, School of Mathematics and Physics, Queens University Belfast, Belfast BT7 1NN, UK; smoutari@yahoo.fr; 5 College of Health and Life Sciences, Aston University, Birmingham B4 7ET, UK

**Keywords:** MM, Barrett, EVO, AS-OCT ray tracing

## Abstract

A comparison of the accuracy of intraocular lens (IOL) power calculation formulae, including SRK/T, HofferQ, Holladay 1, Haigis, MM, Barrett Universal II (BUII), Emmetropia Verifying Optical (EVO), and AS-OCT ray tracing, was performed. One hundred eyes implanted with either the Rayone EMV RAO200E (Rayner Intraocular Lenses Limited, Worthing, UK) or the Artis Symbiose (Cristalens Industrie, Lannion, France) IOL were included. Biometry was obtained using IOLMaster 700 (Carl Zeiss Meditec AG, Jena, Germany) and MS-39 AS-OCT (CSO, Firenze, Italy). Mean (MAE) and median (MedAE) absolute errors and percentage of eyes within ±0.25D, ±0.50D, ±0.75D, and ±1.00D of the target were compared, with ±0.75D considered a key metric. The highest percentage within ±0.75D was found with MM (96%) followed by the Haigis (94%) for the enhanced monofocal IOL. SRK/T (94%) had the highest percentage within ±0.75D, followed by Holladay 1, MM, BUII, and ray tracing (all 90%) for the multifocal IOL. No statistically significant difference in MAE was found with both IOLs. EVO showed the lowest MAE for the enhanced monofocal and ray tracing for the multifocal IOL. EVO and ray tracing showed the lowest MedAE for the two respective IOLs. A similar performance with high accuracy across formulae was found. MM and ray tracing appear to have similar accuracy to the well-established formulae and displayed a high percentage of eyes within ±0.75D.

## 1. Introduction

In modern lens-based surgery, accurate refractive outcomes are expected and increasingly achieved as surgical equipment, biometry, and intraocular lens (IOL) selection improve. The development and introduction of new formulae continues to improve biometry accuracy, with studies showing that the latest generation formulae perform better than previous generations [1,2]. However, there is still no definitive conclusion as to which is currently the best formula.

IOL power calculations have advanced considerably since the early vergence formulae, with progression to include more complex theoretical mathematical derivations and include more patient variables and more mathematical constants to improve the prediction of the effective lens position (ELP) [3].

One such advancement is the Barrett Universal II (BUII) formula [4], which is based upon a theoretical and regression model. This formula has been found to outperform previous generation formulae [1,5]. Furthermore, ray-tracing and artificial intelligence methods are increasingly being used to enhance the performance of IOL power formulae. Ray tracing utilises Snell’s law to calculate the refraction of light through each refractive medium of the eye to predict the postoperative IOL position. Ray tracing can be utilised to calculate an anatomical IOL position rather than the theoretical ELP, such as the Olsen formula [6], which utilises exact and paraxial ray tracings through refractive media, including optics of the IOL, to derive the postoperative position of that lens [3]. Olsen relates the preoperative ACD and lens thickness to the IOL centre to predict the postoperative IOL position [3,6]. With ray tracing, corneal tomography measurements of the anterior and posterior corneal surface elevations are used, in contrast to anterior keratometric measurements in other IOL calculation formulae. Furthermore, higher-order aberrations, like spherical aberration, are taken into account, and ray tracing uses real refractive indices and predicts the postoperative refractive aberrations and retinal image quality [7,8]. The ray tracing approach has been found to provide accurate IOL power calculations with different patient groups [6,8,9,10,11].

MS-39 anterior segment optical coherence tomography (AS-OCT) (CSO, Firenze, Italy) is a new AS-OCT that has been introduced with an inbuilt ray tracing program. Savini et al. [8] investigated the use of MS-39 ray tracing in eyes with a history of myopic excimer laser; however, there are limited published studies on this new AS-OCT ray tracing methodology. Another approach is IOL power calculation through artificial intelligence. Such formulae use the achieved postoperative refractive outcomes to calculate the IOL power required through regression analysis and artificial intelligence. The accuracy is improved further when increasing numbers of postoperative outcomes are incorporated into the artificial intelligence algorithm [3]. Examples of such formulae include the Hill-radial basis function, Ladas Super Formula, or the Kane formula, which uses artificial intelligence with theoretical optics [3,5]. Further to this, machine learning has been introduced for IOL power calculation. The MM formula is a thin lens machine learning formula that utilises an ensemble of regression models to obtain a more accurate prediction of the ELP [12]. A high number of IOL constants are optimally devised, stored, and managed through the MM model [12]. This latest machine learning formula has not been included in any previous comparison study.

Another new formula is the Emmetropia Verifying Optical (EVO), which is based upon the theory of emmetropisation. The EVO uses keratometry, axial length (AL), and anterior chamber depth (ACD), with white to white and lens thickness as optional variables [3]. This formula has been found to be accurate and comparable to other formulae [13].

As outlined, there are various IOL power calculation methodologies available that have advanced from the early vergence formulae to ray tracing and machine learning. Biometry accuracy is improving; however, there is no definitive guidance to determine which IOL calculation methodology to use. More IOL calculation formulae are being released, and not all have been compared. Therefore, the purpose of this study is to evaluate four commonly used vergence formulae, the above-mentioned latest generation vergence formulae, and in particular a machine learning formula (MM formula) and a ray tracing IOL power calculation (MS-39 ray tracing), with which there is a paucity of literature currently. A comparison between this selection of IOL formulae has not previously been completed.

## 2. Materials and Methods

This retrospective study recruited consecutive patients who underwent uneventful cataract extraction surgery or refractive lens exchange and were implanted with either an Artis Symbiose Mid/Plus IOL (Cristalens Industrie, Lannion, France) or a Rayone EMV (Rayner Intraocular Lenses Limited, Worthing, UK) IOL. All patients recruited gave their informed consent for their anonymised data to be analysed and used for audit and publication. All data were unidentifiable. Inclusion criteria were successful biometry and AS-OCT measurements, uneventful surgery with no complications during or after surgery, and refraction 3 months postoperatively. Exclusion criteria were any other ocular pathology or any previous refractive surgery.

Each patient underwent a full preoperative ophthalmological examination. Biometry was completed with the IOLMaster 700 (Carl Zeiss Meditec AG, Jena, Germany) and corneal tomography with the MS-39 AS-OCT (CSO, Firenze, Italy). The IOLMaster 700 was used to select the IOL power received by each patient.

Patients were then assessed 3 months postoperatively, where uncorrected (UDVA) and corrected (CDVA) distance visual acuities were evaluated with logarithmic acuity (logMAR) charts, and manifest refraction was measured by an optometrist. AS-OCT and biometry were repeated postoperatively at this 3-month assessment.

### 2.1. Formulae

The formulae utilised in this study were the SRK/T [14], HofferQ [15], Holladay 1 [16], Haigis [17], MM [12], the BUII [4], EVO, and ray tracing based on AS-OCT. The IOLCon website (https://iolcon.org, accessed on 17 May 2024), which has been previously outlined [18], was used to access the latest constants for the SRK/T, HofferQ, Holladay 1, and Haigis formulae. Many comparison studies have been published, and many utilise optimised constants with zeroing out of the mean prediction error [19,20]. This methodology has been widely accepted; however, it has not been utilised in this study. The IOLCon latest constants were used because the MM formula is a machine learning formula and optimisation of the constants in this manner is not possible; therefore, a direct comparison would not be appropriate. Furthermore, similar to the methodology used in a study by Kenny et al. [21], utilisation of the IOLCon constants allowed a real-life comparison of the performance of formulae because in most ophthalmology clinics these are the constants that are used when deciding which IOL power to implant; therefore, this allows an analysis of actual real-life clinical outcomes.

The MM [12] formula uses thin lens geometric optics, machine learning, and an ensemble-based approach to estimate the ELP. The formula utilises preoperative keratometry readings, AL, and ACD. The authors explain that the formula provides a surgeon-specific and also a self-sustained methodology because the more data available to train the formula, the more “personalized” the IOL estimation [12]. This study utilised 200 eyes implanted with each IOL design to optimise the MM formula. The IOLMaster 700 preoperative keratometry readings, AL, and ACD and the 3-month postoperative refractive data were utilised to optimise the MM formula for each separate lens design included in this study. This optimised MM formula was then applied to the study cohort for both the Rayone EMV and the Artis Symbiose IOLs to calculate the predicted spherical equivalent (SE) target for the actual implanted IOL.

The BUII formula is a well-recognised formula and has been shown to have good accuracy in different patient groups [2,22,23]. The derivation of the formula is unpublished, but it is based upon a theoretical model eye and retains the correlation between AL and corneal shape to ACD [3].

The EVO formula is a thick lens vergence formula and is a new formula that is unpublished. This formula creates an “emmetropia factor” for each eye and considers the optical parameters for different IOL geometry and powers [13]. The formula is accessed through an online calculator (https://www.evoiolcalculator.com (accessed on 17 May 2024), and the required parameters are keratometry readings, AL, and ACD.

For both the BUII and EVO formulae, this study used a recently published web scraping method [24] to allow automatic data input into the online calculators to predict the required IOL power outputs and corresponding spherical equivalent (SE) refractions. The constants used were 119.74 for the Artis Symbiose IOLs and 118.6 for the Rayone EMV, which were taken from the IOLCON website (https://iolcon.org).

Ray tracing biometry was completed using the MS-39 AS-OCT and the inbuilt ray tracing software, which has previously been described by Savini et al. [8]. The AL from the IOLMaster 700 and the corresponding A constants, 119.7 for the Artis Symbiose IOLs and 118.6 for the Rayone EMV, were entered into the ray tracing program. The corresponding SE prediction for the implanted IOL was recorded. A constant of 119.7 for the Artis Symbiose IOL was used for the ray tracing AS-OCT because constants are only available to one decimal place.

### 2.2. Methods of Formula Comparison

The prediction error was calculated as the difference between the postoperative 3-month SE refraction and the predicted SE for each formula. The mean absolute prediction error (MAE) and the median absolute prediction error (MedAE) were calculated for each formula and IOL, and the percentage of eyes within ±0.25 D, ±0.50 D, ±0.75 D, and ±1.00 D were compared across the different formulae. The key metric utilised in this study was accuracy of the mean prediction error within ±0.75 D. This was selected because residual SE refractive error above 0.75 D is quite often the threshold for requirement for further refractive surgical intervention, such as laser enhancement surgery. Therefore, comparison of the accuracy to within ±0.75 D would allow vital clinically relevant information regarding the studied formula for these presbyopia-correcting IOLs.

### 2.3. Statistical Analysis

The statistical software SPSS version 26.0 (IBM, Armonk, NY, USA) was used for data analysis. The Kolmogorov–Smirnov test was used to assess the normality of the data. To determine the deviation of the mean prediction error from zero, the one sample *t*-test was completed. The Friedman’s test with Bonferroni correction was used for pair-wise comparison of absolute errors. The Cochrane Q test Bonferroni correction was performed to compare the percentages of eyes within ±0.25 D, ±0.50 D, ±0.75 D, and ±1.00 D of the absolute prediction error. A *p* value < 0.05 was considered to be statistically significant.

Following the methods outlined by Goodall et al. [25], a sample size of 41 eyes was required for an 80% statistical power. The standard deviation (SD) of the MAE was determined to be 0.4, which was motivated by insights gained through previous studies. A 0.25 D difference in MAE was considered to be clinically significant as determined by clinical experience. For all statistical analysis, the level of significance was a *p* value less than 0.05.

### 2.4. Intraocular Lens

The Artis Symbiose Mid IOL and Artis Symbiose Plus IOL are complementary continuous-phase multifocal IOLs to be used in combination. The Artis Symbiose IOL combination provides continuous addition from 1.5 dioptres (D) to 3.75 D, where the Artis Symbiose Mid IOL provides superior intermediate vision with a maximum at 1.75 D and the Artis Symbiose Plus IOL provides superior near vision with a maximum at 3.25 D. In combination, the IOLs provide a range of focus from 40 to 90 cm without compromising distance vision through “modulated profiles” technology, which creates asymmetrical depth of field with complementarity in binocular vision [26]. The IOLs have a diffractive profile to give continuous through-focus phase near to intermediate vision [26].

The IOL has a 6 mm optic diameter and a 10.79 overall length. There is a diffractive profile on the anterior surface with the diffractive rings in the central 4.2 mm of the optic, and outside this zone, the optic is purely refractive. The IOL is aspherical with −0.23 μm spherical aberration. It is made of hydrophobic material with four closed-loop haptics and is available +10.0 to +35.0 D.

The Rayone EMV is an enhanced monofocal IOL that utilises increased positive spherical aberration to enhance depth of focus, achieved through discrete local power changes in the centre of the IOL optic [27]. The IOL is designed to be used with a monovision approach, with one eye targeted for emmetropia and the other with a myopic offset. The manufacturers state that the IOL provides up to 1.5 D range of focus with an emmetropic target, and with a myopic offset of 1.0 D, a depth of focus of 2.25 D is produced. The unique positive spherical aberration design enhances the depth of focus and provides a smoother transition between eyes. The periphery of the optic behaves as an aberration-neutral IOL [27].

The IOL material is hydrophilic acrylic, and the optic diameter is 6.00 mm with an overall length of 12.5 mm. The available powers are +10.0 to +30.0 D in 0.50 D increments.

### 2.5. Surgical Technique

The surgeries were completed by two experienced surgeons (JM and NM). The surgeries were performed under Sub-Tenon anaesthesia with standard clear corneal phacoemulsification, and the foldable IOL was inserted through a 2.4 mm incision. An anterior capsulorhexis of 5.5 mm was defined, and implantation of the IOL was into the capsular bag.

## 3. Results

The study included 100 eyes of 61 patients. Fifty eyes were implanted with an enhanced monofocal IOL and 50 eyes with a multifocal IOL. Table 1 outlines the patient demographics. Table 2 presents the IOL constants for the two IOL designs. 

### 3.1. Enhanced Monofocal IOL

Table 3 outlines the prediction accuracy to within ±0.25 D, ±0.50 D, ±0.75 D, and ±1.00 D. There was no significant difference between the formulae for accuracy to within ±0.25 D, ±0.50 D, ±0.75 D, and ±1.00 D of the target. The MM formula showed the highest percentage of eyes within ±0.75 D with 96%, followed by Haigis with 94%.

The HofferQ, Holladay 1, Haigis, MM, EVO, BUII, and ray tracing showed a statistically significant difference from zero for the mean refraction prediction error (Table 4).

Table 3 outlines the mean prediction error MAE, where the lowest MAE was found with the EVO and Haigis formulae. No statistically significant difference was found when comparing the MAE across the formulae. The lowest MedAE was found with the EVO formula (Table 3). The maximum absolute error was produced by BUII and HofferQ (Table 3).

### 3.2. Multifocal IOL

There was a statistically significant difference between the formula for accuracy within ±0.25 D. Pairwise post hoc analysis revealed significant differences in the following comparisons: ray tracing vs. Holladay 1 (*p* = 0.019), ray tracing vs. HofferQ (*p* = 0.009), ray tracing vs. Haigis (*p* = 0.004), ray tracing vs. SRK/T (*p* = 0.002), and ray tracing vs. BUII (*p* < 0.001). There was no significant difference across formulae within ±0.50 D, ±0.75 D, and ±1.00 D accuracy to the target. The SRK/T, followed by the Holladay 1, MM, BUII, and ray tracing, had the highest percentage of eyes within ±0.75 D (Table 3).

The SRK/T (*p* = 0.041), Haigis (*p* = 0.033), and BUII (*p* = 0.023) formulae showed a statistically significant difference from zero for the mean prediction error (Table 4).

The lowest MAE was found with AS-OCT ray tracing, followed by Holladay 1 (Table 3). There was no statistically significant difference in MAE between formulae. The lowest MedAE was found with the ray tracing formula, and the Haigis and HofferQ formulae had the highest error (Table 3).

## 4. Discussion

This study sought to compare the accuracy of the latest generation of IOL formulae and compare different IOL power calculation methodologies. The BUII and the EVO formula have been shown to produce good biometry accuracy [28,29], and both are freely available as online calculators, and the BUII is also available on the IOLMaster 700. Ray tracing and artificial intelligence are the latest technologies to be used in IOL power calculations. To our knowledge, there is no comparison of ray tracing using the MS-39 AS-OCT to other formulae, and there is no comparison of the MM machine learning formula to other commonly used formulae. A study has reported the accuracy of MS-39 AS-OCT ray tracing in eyes with a history of myopic excimer laser surgery [8]. The formulae were selected because they are readily available through online calculators, IOLMAster 700, MS-39 AS-OCT, or published methodology, and they have not previously been compared.

The concept of optimisation leading to a zero mean error is widely utilised in biometry studies; however, this methodology was not used in this study. This study sought to compare various different formulae, including formulae based on artificial intelligence or machine learning models. The methods of optimising the mean error to zero cannot be used when comparing machine learning or artificial intelligence formulae because it will overfit and give unfair advantage to these types of formulae. The main recognised methods to compare and contrast machine learning formulae use “k fold cross validation”, which requires significant computing techniques to repeat sampling to produce recurrent new training and test sets. However, this is not possible within the confines of such a study where some of the formulae being used and compared have not been published and, therefore, cannot be computed. Additionally, zeroing of the mean error was not followed in this study because optimising constants to achieve a mean error of zero for one dataset will not produce a mean error of zero when applied to another set of data, and, therefore, it gives limited clinical information. The use of a training set to optimise formula constants, which is then applied to the test/study set, would produce more real-life outcomes and, therefore, more meaningful analysis. Therefore, in this retrospective study, the actual prediction error calculated from the IOLCon constants and the achieved postoperative refractive outcome were utilised. This allowed analysis of the real-life refractive outcomes for each case. This also allowed for direct comparison between the MM formula, which is a machine learning methodology. Optimisation of the IOL constants to adjust the mean prediction error to zero [19,20] would skew the results of the other formula based upon IOL constants because of optimisation to the specific test dataset. Many studies have used this methodology to compare the MAE and accuracy to the target. However, this does not allow real-life comparison because the outcomes are optimised for the specific test set and, therefore, can show falsely increased accuracy of outcomes and not real-life clinical outcomes. Additionally, this study sought to use accuracy to within ±0.75 D as a key metric because cases with residual SE refractive error greater than 0.75 D often require further refractive interventions. Special attention to ±0.75 D would give real-life clinically relevant information, which would inform future clinical decisions within the clinic. Optimisation of constants to zero out the prediction error and then comparison of the MAE frequently do not result in statistically significant differences or even clinically significant differences. 

In this current study, the prediction error was within ±0.50 D in at least 70% of eyes and within ±1.0 D in at least 94% of eyes for each formula (Table 3), which is superior to previously published benchmarks [30]. There were no significant differences across each formula for accuracy within ±0.25 D, ±0.50 D, ±0.75 D, and ±1.00 D. Similar results were found with the multifocal IOL in this study, again with superior results of eyes within ±0.50 D and ±1.00 D compared to benchmarks [30], with no significant difference across each formula in regards to percentage accuracy. The MM formula showed the highest percentage of eyes within ±0.75 D, with 96% of eyes achieving this, followed by the Haigis formula, with 94% for the enhanced monofocal IOL (Table 3). For the multifocal IOL, the SRK/T, followed by Holladay 1, MM, BUII, and ray tracing had the highest percentage within ±0.75 D. All formulae showed high accuracy to this threshold of ±0.75 D, with the newly developed MM formula and ray tracing showing comparable and high accuracy. 

Various studies outline the accuracy of vergence formulae and the newer generation formulae [13,31,32]; however, to our knowledge, there are no studies that include a comparison to the MM [12] formula and AS-OCT ray tracing. Therefore, this study sought to compare the MM formula and AS-OCT to the formulae that have been previously reported.

The MM formula had an MAE of 0.34 and 0.37 (Table 4), and 78% and 68% of eyes were within ±0.50 D for the two IOL designs. This is superior to another comparison study where the best performing formula in terms of MAE was the BUII, with a value of 0.385, and 72% of eyes were within ±0.50 D [1]. Also, the MedAE is 0.27 and 0.32 with a maximum error of 1.21 and 1.20 for the two IOL designs (Table 4). Compared to another study [29] of six modern formulae, the MedAE are similar; however, the maximum error is significantly lower in this current study with the MM formula. 

Ray tracing biometry utilises the anterior and posterior corneal surfaces instead of simply keratometric readings and is being used with regular and irregular corneas. The advantage of ray tracing has been reported to reduce error due to being based upon physical measurements, using real refractive indices, and not following thin lens formulae [8]. The MS-39 has been shown to be accurate in eyes with previous myopic excimer laser surgery [8]; however, there are no comparison studies to other formulae in normal corneas. In this current study, as previously outlined, there was no significant difference to the other studied formulae in terms of MAE. Furthermore, there were no significant differences in accuracy to the target compared to other formulae. The ray tracing methodology did show a significantly higher percentage of eyes within ±0.25 D compared to other formulae (Table 3) for the multifocal IOL. However, there was no significant difference between formulae within ±0.50 D, ±0.75 D, and ±1.00 D. This is a promising finding due to the importance of accuracy to the predicted target in multifocal IOL selection. Further studies with increased numbers will investigate this further. This study does display that ray tracing with the MS-39 is comparable to other readily available formulae; however, further investigation is required to determine if it is truly beneficial to use this methodology as it is more time-consuming compared to other modern formulae incorporated into current biometry machines.

A limitation of this current study is the relatively small sample size of the two different IOL designs, which meant that comparison between different axial lengths was not completed, and that both eyes of the same patient were included in this study, which may impact inter-eye correlation Further studies with greater numbers would increase the understanding of biometry accuracy with these IOLs and the impact of axial length upon biometry accuracy. Furthermore, comparison to other biometry accuracy papers is limited due to different methodologies because many papers optimise constants and zero out the prediction error. This study did not follow this methodology to present real-life outcomes utilising up-to-date IOLCon constants, therefore allowing for direct comparison between the different formulae and, in particular, to gain an understanding of how the newly developed MM formula and ray tracing compare to other traditional and latest generation formulae.

## 5. Conclusions

In conclusion, this study showed high similar accuracy across the formulae for both IOL presbyopia-correcting designs. The newly developed MM formula and MS-39 AS-OCT biometry showed comparable outcomes compared to other well-established formulae, and high accuracy to within ±0.75 D would be expected with both new formulae for the use with presbyopia-correcting IOLs. In order to enable clinically relevant comparisons between increasingly complex formulae, which utilise differing methodologies, it would make sense in the future to try to initially utilise representative training sets within the clinic setup to enable optimisation of each formulae and then subsequently compare the formulae utility upon separate test sets but without any further “constants” optimisation.

## Figures and Tables

**Table 1:** Patient demographics.

Parameter	Values
Mean Age (years) ± SD, range	59 ± 6.96 (42 to 79)
Female gender, n (%)	34 (55.7)
Mean axial length (mm) ± SD, range	23.45 ± 1.15 (21.3 to 26.33)
Mean anterior chamber depth (mm) ± SD, range	3.16 ± 0.37 (2.05 to 4.02)
Mean Keratometry (D) ± SD, range	43.17 ± 1.26 (39.88 to 45.90)
Mean IOL power (D) ± SD, range	22.5 ± 3.61 (12 to 30)
Mean postoperative spherical equivalent ± SD, range	−0.28 ± 0.67 (−2.13 to 1.25)
Mean postoperative cylinder power ± SD, range	−0.44 ± 0.30 (−1.25 to 0)

D = dioptre; SD = standard deviation.

**Table 2:** IOL constants.

	EMV	Artis Symbiose
Barrett	118.6	119.74
EVO	118.6	119.74
Haigis	a0 1.044	a0 0.088
	a1 0.4	a1 0.233
	a2 0.1	a2 0.2
HofferQ	5.32	6.095
Holladay 1	1.56	2.295
MM		
Ray tracing	118.6	119.7
SRK/T	118.6	119.74

**Table 3:** a. Results of Rayner EMV IOL power calculation using the different formulae. b. Results of Artis Symbiose IOL power calculation using the different formulae.

Formula	MAE	MedAE	Max Error	% within ±0.25 D	% within ±0.5 D	% within ±0.75 D	% within ±1.00 D
a							
Barrett Universal II	0.40	0.33	1.29	34	74	86	96
EVO	0.31	0.22	1.28	56	82	90	96
Haigis	0.32	0.25	1.12	50	82	94	96
HofferQ	0.39	0.32	1.29	40	72	84	94
Holladay 1	0.35	0.31	1.16	46	80	90	96
MM	0.34	0.27	1.21	44	78	96	98
Ray tracing	0.38	0.36	1.25	34	70	90	96
SRK/T	0.34	0.26	1.24	48	78	92	96
b							
Barrett Universal II	0.40	0.37	1.13	30	72	90	98
EVO	0.37	0.30	1.01	42	78	86	98
Haigis	0.40	0.34	1.30	34	72	82	98
HofferQ	0.41	0.32	1.30	36	68	84	92
Holladay 1	0.36	0.30	1.06	38	74	90	98
MM	0.37	0.32	1.20	42	68	90	98
Ray tracing	0.34	0.24	1.17	56	74	90	94
SRK/T	0.38	0.33	0.97	32	76	94	100

MAE = mean absolute error; Max error = maximum absolute error; MedAE = median absolute error.

**Table 4:** Prediction errors for each formula and *p* values from the one sample *t*-test used to determine significant difference from zero.

Formula	EMV		Artis Symbiose	
	PE ± SD	*p* Value	PE ± SD	*p* Value
Barrett Universal II	−0.28 ± 0.39	<0.001	0.15 ± 0.44	0.023
EVO	0.23 ± 0.36	<0.001	−0.06 ± 0.45	0.348
Haigis	−0.16 ± 0.38	0.002	−0.15 ± 0.49	0.033
HofferQ	−0.26± 0.42	<0.001	−0.12 ± 0.51	0.099
Holladay 1	−0.21 ± 0.40	<0.001	0.01 ± 0.45	0.926
MM	−0.22 ± 0.36	<0.001	−0.04. ± 0.45	0.538
Ray tracing	−0.14 ± 0.46	0.036	0.03 ± 0.46	0.601
SRK/T	−0.13 ± 0.42	0.30	0.13 ± 0.42	0.041

PE = prediction error; SD = standard deviation.

## Data Availability

The datasets used and/or analysed during the current study are available from the corresponding author on reasonable request.
